# Evaluation of diflubenzuron–verapamil combination strategy for eco-safe management of *Aedes aegypti*

**DOI:** 10.3389/fphys.2024.1476259

**Published:** 2024-12-16

**Authors:** Manu Sankar, Divya Yadav, Sarita Kumar

**Affiliations:** Department of Zoology, Acharya Narendra Dev College, University of Delhi, New Delhi, India

**Keywords:** diflubenzuron, enzyme expression, mosquito management, non-targets, synergism, verapamil

## Abstract

**Introduction:**

*Aedes aegypti*, the vector of multiple arboviral diseases, is a prime health concern worldwide. The surge in *Aedes-*borne diseases emphasizes the urgent need for efficient vector control measures. Synthetic pesticides used traditionally, however, present environmental concerns and issues like resistance development, causing the use of higher chemical doses. Hence, alternate interventions like the use of insect growth regulators (diflubenzuron; DFB) show promise because of their unique mechanism of action and environmental safety. Nevertheless, mosquitoes have the potential to develop resistance to any chemical. Thus, the present study investigates the use of DFB in combination with verapamil (DFB-V; 1:10) as a possible mosquito intervention measure.

**Methods:**

The effects of both DFB and DFB-V were assessed on the larval development, adult emergence and expression of detoxification enzymes, non-specific esterases, glutathione S-transferase (GST), acetylcholinesterase (AChE), and monooxygenases in laboratory-reared (AND-*Ae. aegypti*) and wild-caught (GVD-*Ae. aegypti*) strains of *Ae. aegypti*. The effects on the survival of non-target organisms were also investigated.

**Results:**

The investigations showed that DFB-V treatment of the *Ae. aegypti* fourth instars caused a 1.16–1.37 fold higher adult emergence suppression than DFB alone, reducing the IE_50_ values. The DFB treatment increased β-esterases, AChE, and monooxygenases but reduced the GST and α-esterase levels. The effects enhanced with the use of DFB-V, causing a significant decrease in α-esterase (7.7-fold) and an increase in monooxygenases (7.8-fold) (*p* < 0.05) in AND-*Ae. aegypti* compared to the wild-caught strain. The variation in enzyme levels in the two strains may be due to the stress caused by insecticides of different chemical natures used in the fields. No negative effects were observed on the non-target organisms—*Gambusia affinis*, *Mesocyclops thermocyclopoides*, and *Paramecium tetraurelia*.

**Conclusion:**

The studies showed the growth regulatory efficacy of DFB and probable role of GST and α-esterases in increasing the effects of DFB when synergized with verapamil. Further, the DFB-V combination did not result in any significant negative effects on the non-target organisms ascertaining its safe use. This is the first report unraveling the effects of the DFB–verapamil combination on the defense mechanism of *Ae. aegypti.* Further studies may assist in developing focused and eco-safe plans for managing *Ae. aegypti* populations effectively.

## 1 Introduction

*Aedes aegypti* is a cosmopolitan vector transmitting various human arboviral diseases such as dengue, Zika, chikungunya, and yellow fever. Though prevalent worldwide, it is predominant in tropical and subtropical regions, situated between 35°N and 35°S latitudes, because of the favorable climatic conditions of temperature and humidity. Over recent decades, *Aedes-*borne diseases have increased, accounting for severe health hazards and loss of human lives. Among these, dengue fever is the most rapidly spreading illness, spanning a geographic range of Southeast Asia to the United States and Western Pacific nations. As per reports, roughly 70% of the dengue cases have been reported from Asia, presumably because of the favorable climatic conditions (WHO, 2024). The lack of operative vaccines and effective medications against *Aedes-*borne illnesses, except for yellow fever, has made the situation grave. Hence, the only tactic to alleviate these illnesses is managing the *Ae. aegypti* population below the threshold limits by employing various intervention measures in and around human settlements.

The mosquito population has been habitually tackled by eliminating breeding sites, avoiding human-mosquito contact by use of mosquito repellents, and killing various stages of their life cycle via employment of synthetic chemicals in the form of spray, dust, mosquito coils, etc. (Bharati and Saha, 2018). However, the extensive and intermittent usage of these chemicals and the favorable selection of more resilient individuals in natural field populations have resulted in the development of resistance in mosquitoes, aggravating the associated issues (Kumar et al., 2002; 2004; Borase et al., 2013). Apart from these, the harmful effects of these chemicals on the environment and human health have diverted the attention of health workers and vector control programmers towards alternative and relatively safer insecticides. Among these insecticides, insect growth regulators (IGRs), the fourth-generation insecticides, are considered a viable and sustainable option to be used against mosquitoes. They impede the growth and development of insects and reduce reproductive fitness by either inhibiting the synthesis of cuticular or peritrophic matrix chitin or interfering with the endocrine functions in the insects (Doucet and Retnakaran, 2012). Furthermore, these compounds are deemed safe for the environment due to their specificity to the target organisms, posing minimal risks to non-target and beneficial biota (Zibadee et al., 2011).

Diflubenzuron (DFB), also known as dimilin, is a benzoylphenyl urea chitin synthesis inhibitor that obstructs the growth and development of insects. It is a commonly used mosquito larvicide that has been approved by the World Health Organisation due to its efficacy and environmental safety (Sankar and Kumar, 2023). The permitted use of DFB in drinking water at the recommended dosage of ≤0.25 mg/L has resulted in its frequent use in mosquito management programs. Various studies have demonstrated the control potential of diflubenzuron against different species of mosquitoes. These studies have shown that DFB caused effective inhibition of ecdysis in *Aedes* sp. larvae with residual activity (Chen et al., 2008), suppression of the *Culex pipiens* population (Pešić et al., 2022), and successful prevention of the *Anopheles* and *Culex* larval emergence leading to ∼80% reduction in larval density (Eltahir et al., 2018).

Nonetheless, mosquitoes possess the capability to develop resistance to different xenobiotics through various mechanisms, such as reduced cuticular penetration of insecticide, increased levels of detoxifying enzymes, and target-site insensitivity (Yao et al., 2017; Karunaratne et al., 2018). The development of DFB resistance has been recognized in *Cx. pipiens* through a variety of mechanisms, evidenced by changes in cuticle thickness, chitin content, and chitin-synthase 1 gene overexpression in the resistant strains (Belinato and Valle, 2015; Porretta et al., 2019; Guz et al., 2020; Lucchesi et al., 2022). In Italy, *Cx. pipiens* strain developed 32.5-fold DFB resistance after 2 years of intensive application, which increased dramatically to 128-fold after another year of application (Grigoraki et al., 2017). The studies revealed the occurrence of I1043M and I1043L mutations in the chitin synthase gene of the resistant population. In addition, the biochemical detoxification of toxins is one of the most significant and rapidly developed mechanisms to provide immunity in insects (Enayati et al., 2005). A few studies have also indicated the possible role of ATP-binding cassette (ABC) transporters in imparting diflubenzuron resistance (Porretta et al., 2008). It has been shown that efflux transporter, P-glycoproteins (P-gps), actively transport toxic molecules out of the cells, reducing the concentrations that reach the target and thus leading to resistance (Epis et al., 2014). One of the inhibitors of P-gp transporters, verapamil, is regarded as a DFB synergist with the potential to reduce the development of DFB resistance in insects (Kang et al., 2016).

Our preliminary studies have shown the considerable efficacy of the diflubenzuron-verapamil combination (DFB-V; 1:10) in enhancing the effects of DFB. The present study aimed to evaluate the effect of diflubenzuron and a diflubenzuron-verapamil combination (1:10) on the adult emergence, total proteins, and levels of detoxification enzymes of early fourth instar larvae of two strains of *Ae. aegypti;* laboratory-reared insecticide-susceptible (AND-*Ae. aegypti*) and wild-caught (GVD*-Ae. aegypti*). The inhibition of adult emergence and titers of different enzymes, glutathione-S transferase, acetylcholinesterase (AChE), non-specific esterases (α and β), and CYP450 monooxygenases, were determined in both the strains after treatments. Along with these, the effects of DFB and DFB-V were also assessed on the non-target organisms: *Gambusia affinis*, *Mesocyclops thermocyclopoides*, and *Paramecium tetraurelia.* These studies ascertain the possible use of verapamil with DFB as a synergist and could help devise an environmentally friendly strategy in vector control programs.

## 2 Materials and methods

### 2.1 Rearing of mosquitoes

The culture of *Ae. aegypti* had been maintained in a well-established rearing laboratory at Acharya Narendra Dev College, New Delhi, India. The mosquitoes were reared under controlled conditions of 28°C ± 1°C, 80% ± 5% relative humidity, and a 14:10 L:D photo-regime. The larvae were hatched in dechlorinated water taken in enamel trays (15 in × 15 in) and fed upon a 3:1 (w/w) mixture of dog biscuit and dry yeast powder (Warikoo and Kumar, 2013) for optimal development. Adults were allowed to emerge in the cloth cages containing water-soaked raisins in a Petri dish for feeding (Warikoo and Kumar, 2013). Female adults were provided periodic blood meals from albino rats sourced from the rearing house of the Department of Zoology set up for the purpose. The eggs were gathered on wet Whatman paper strips and hatched in dechlorinated water.

### 2.2 Chemicals required

The technical-grade diflubenzuron (DFB) with a purity level of 98.0% (CAS No. 35367-38-5) and verapamil with ≥99.0% purity (CAS No. 152-11-4) were obtained from Sigma-Aldrich, India.

### 2.3 Strains of *Ae*. *aegypti* used for investigations

(a) Laboratory-reared insecticide-susceptible strain (AND-*Ae. aegypti*): The strain was obtained in 2009 from the International Centre for Genetic Engineering and Biotechnology, New Delhi, India, and maintained in the laboratory without the selection pressure of any insecticide.
(b) Govindpuri strain of *Ae. aegypti* (GVD*-Ae. aegypti*): Larvae were collected from the fields of the Govindpuri locality of Southeast Delhi, India (28.534°N, 77.265°E) and brought to the laboratory for investigations.

### 2.4 Adult emergence inhibition studies with diflubenzuron and diflubenzuron-verapamil combination (DFB-V)

The adult emergence inhibition potential of DFB and DFB-V was estimated in accordance with the WHO protocol (WHO, 2005). The combination of DFB–verapamil was prepared in a 1:10 ratio selected after preliminary investigations. The early fourth instar larvae of *Ae. aegypti* were treated with a series of DFB/DFB-V concentrations ranging from 0.0625 µg/L to 16 µg/L for 24 h in three replicates. In each replicate, a total of 20 larvae were treated with a homogenous mixture of 1 mL of a specific concentration of DFB/DFB-V and 199 mL of distilled water. The surviving larvae were reared to record the adult emergence. Control sets were run simultaneously. The percent inhibition of adult emergence (IE%) was calculated as follows (Equation 1):

$$\text{IE }\%=100-\left\{\frac{\text{T X}\;100}{C}\right\}$$ (1)

where T represents the percentage of adult emergence in treated sets, and C represents the percentage of adult emergence in the control set. The data were subjected to probit mortality-regression analysis by the SPSS 19.0 program, and IE_50_ dosages of diflubenzuron were computed along with other statistical parameters.

The synergistic potential of verapamil was calculated as per the formula (Equation 2) given below:

$$\text{Syn}{\text{ergistic}\;\text{factor}\;\left(\text{SF}\right)=\text{IE}}_{50}\;\text{dosage}\;\text{of}\;\text{DFB}\;\text{alone}\;\;/{\;\text{IE}}_{50}\;\text{dosage}\;\text{of}\;\text{DFB}\;-\;\text{Verapamil}.$$ (2)

### 2.5 Biochemical characterization of detoxification enzymes

A total of fifty (50) early fourth instars of both the strains of *Ae. aegypti* were treated with the diflubenzuron alone and synergized DFB (DFB-V) at respective IE_50_ dosages. Twenty surviving larvae were randomly selected after 24 h of treatment and biochemically characterized for proteins and detoxifying enzymes using the standard WHO methodology (WHO, 1998), with a few modifications (Kona et al., 2018). Concurrent control assays were carried out.

#### 2.5.1 Preparation of larval homogenate

Individually treated larva of each strain was homogenized in 200 µL of ice-cold autoclaved water, using a mini-homogenizer. Each larval homogenate was centrifuged at 4°C for 30 s at 17,000 × *g*. The supernatant was used to estimate proteins, glutathione S-transferase (GST), CYP450 monooxygenases, and non-specific esterases (α-esterases and β-esterases). The quantification of acetylcholinesterase was performed using the crude homogenate. The assays were conducted in three replicates. Each replicate consisted of 20 larvae, and each replicate was assayed twice.

#### 2.5.2 Total proteins

Protein estimation in larvae of both the strains of *Ae. aegypti* treated with DFB or DFB-V was carried out using Bradford’s (1976) methodology. The supernatant from each treatment (10 µL) was pipetted into a microtiter plate to which 300 µL of the Bio-Rad protein reagent was added. The homogenate was replaced in blank and standard with water and bovine serum albumin (BSA), respectively. After incubation for 5 min, the plate was read at 570 nm using an ELISA plate reader. The protein standard curve was plotted, and the total proteins in the larva were determined in mg/mL.

#### 2.5.3 GST activity

A mixture of 50 µL of 2 mM GSH (reduced glutathione) and 50 µL of 1 mM CDNB (1-chloro-2,4-dinitrobenzene) was taken in a microtiter plate and supplemented with the 20 µL of the larval homogenate supernatant. The absorbance was measured at 340 nm every minute for continuous 5 minutes (Brogden and Barber, 1990). The enzyme kinetics were computed, and GST activity was calculated as mol/min/mg of protein.

#### 2.5.4 Non-specific esterase titers

10 µL of the homogenate supernatant of *Ae. aegypti* larvae after each treatment was taken in a microtiter plate and mixed with 200 µL of 3 mM solution of either α-naphthyl acetate or β-naphthyl acetate for respective α-esterase and β-esterase quantification. Subsequent to incubation for 15 min, a volume of 50 µL of freshly made 6.3 mM fast blue stain solution was added to each, which resulted in the color change. The absorbance was recorded at 570 nm (Brogden and Dickinson, 1983), and the esterase activity was calculated as nmol of naphthol/min/mg of protein. The standards for calculating α-esterase and β-esterase activity were run with corresponding α-naphthol or β-naphthol.

#### 2.5.5 CYP450 monooxygenase levels

A 20 µL aliquot of the larval homogenate supernatant was taken in a microtiter plate and mixed with 80 µL of 0.625 M potassium phosphate buffer (pH 7.2). It was then added to 200 µL of a solution that contained one part of 0.25 M sodium acetate buffer (pH 5.0) and three parts of 8 mM methanolic solution of tetramethyl benzidine (TMBZ). Subsequently, 25 µL of 0.88 M hydrogen peroxide was added to it, and absorbance was measured at 650 nm after incubation for 10–15 min at ambient temperature. The monooxygenase activity was expressed as mmol/mg of protein.

#### 2.5.6 Inhibition in AChE activity

Two replicates of 25 µL of the crude larval homogenate, placed in the microtiter plate, were supplemented with 145 µL of 0.017 M Triton X-100 and 10 µL of 0.01 M dithiobis 2-nitrobenzoic acid (DTNB). One replicate was mixed with 25 µL of 0.01 M acetylthiocholine iodide (ASCHI), while the other was supplemented with 25 µL of 0.01 M ASCHI + 0.1 M propoxur (500:1). The absorbance was measured at 405 nm after an incubation period of 1 h (Brogden and Barber, 1987).

The endpoint of the reaction was computed by dividing the AChE+propoxur activity by the AChE alone activity. The percent inhibition of acetylcholinesterase was calculated by the formula [100 − (100% × Endpoint)].

#### 2.5.7 Statistical analysis

The Kolmogorov–Smirnov tests using SPSS 19 software were performed to check the normality of enzyme activities. The data obtained with different treatments were statistically analyzed by ANOVA (single-way variance analysis). Tukey’s all-pairwise multiple comparison test was used to compare the means to determine the statistical significance of data at *p* < 0.05.

### 2.6 Effect on non-target organisms

Three non-target organisms, *G. affinis*, *M. thermocyclopoides*, and *P. tetraurelia*, were collected from pond water in the South Delhi region of India. Care was taken to collect the active organisms in good health and of similar size. Each organism was treated with respective IE_50_ dosages of DFB and DFB-V for 24 h computed against *Ae. aegypti* fourth instar larvae. The organisms were added to a mixture of 249 mL of water and 1 mL of treatment dosage. *G. affinis* were treated in groups of 5, while *M. thermocyclopoides* and *P. tetraurelia* were treated in groups of 20 each. The effect of treatment was observed on the survival and morphological alteration of each organism. Each assay was carried out in three replicates. The control sets were run in parallel.

## 3 Results

### 3.1 Adult emergence inhibition studies

**FIGURE 1:**
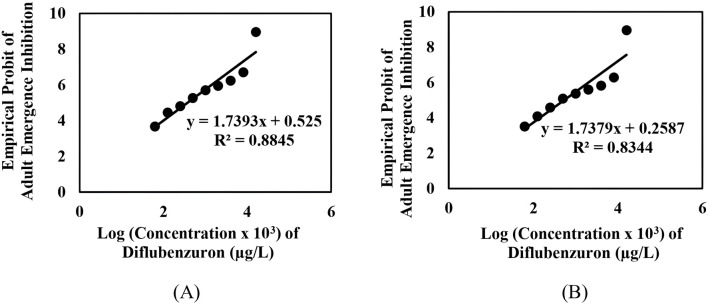
Inhibition of adult emergence in the early fourth instar larvae of *Ae. aegypti* when treated with different concentrations of diflubenzuron: **(A)** insecticide-susceptible, AND-*Aedes aegypti* strain, and **(B)** wild-caught, GVD-*Aedes aegypti* strain.

**FIGURE 2:**
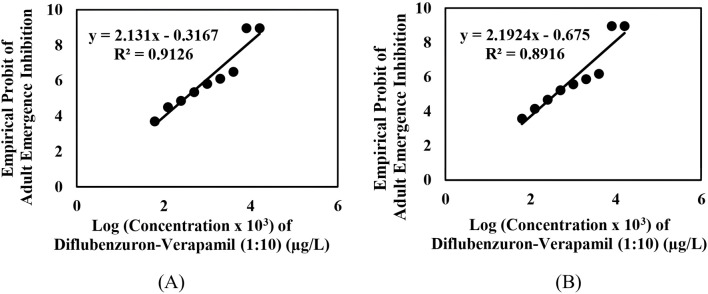
Inhibition of adult emergence in the early fourth instars of *Aedes aegypti* treated with different concentrations of diflubenzuron–verapamil (1:10): **(A)** insecticide-susceptible, AND-*Ae. aegypti* strain, and **(B)** wild-caught, GVD-*Ae. aegypti* strain.

The adult emergence inhibition studies with DFB and DFB-V (1:10) against *Ae. aegypti* larvae showed dose-dependent efficacy; enhanced effects were obtained with DFB-V (Table 1; Figures 1, 2). Treatment of the *Ae. aegypti* fourth instars with DFB suppressed the adult emergence by 9.1%–100% with complete suppression at 16.0 µg/L, while DFB-V could inhibit the emergence completely at 8.0 µg/L. The DFB-V caused 1.16 and 1.37-fold higher suppression in laboratory-reared and wild-caught strains, respectively, than only DFB.

**TABLE 1:** Inhibition of adult emergence from the early fourth instar larvae of *Aedes aegypti* treated with diflubenzuron and diflubenzuron–verapamil (1:10).

Treatment	IE_50_ concentration (µg/L) ± SEM	Slope ± SEM	χ^2^ (df)	*p* value	Synergistic factor (SF)
AND-*Ae. aegypti*					
Diflubenzuron alone	0.37 ± 0.0017 (0.31–0.45)	1.739 ± 0.865	5.576 (6)	0.472	1.156
Diflubenzuron - verapamil (1:10)	0.32 ± 0.0288 (0.27–0.38)	2.131 ± 0.103	3.739 (5)	0.558	
GVD-*Ae. aegypti*					
Diflubenzuron alone	0.63 ± 0.0230 (0.52–0.75)	1.737 ± 0.807	7.728 (6)	0.296	1.369
Diflubenzuron - verapamil (1:10)	0.46 ± 0.0230 (0.38–0.54)	2.192 ± 0.994	4.858 (5)	0.433	

IE values computed by probit mortality-regression analysis using SPSS v. 19 software. SEM: Standard error of the mean. IE_50_ = the concentrations that inhibit 50% of adult emergence. χ2 = chi-square. df = degree of freedom.

### 3.2 Total protein reserves

The larvae of AND-*Ae. aegypti* had 2.44-fold (*p* < 0.05) higher protein content compared to the larvae of GVD*-Ae. aegypti* strain. The larval treatment with IE_50_ dosages of DFB caused insignificant changes in the protein content by 1.02-fold (*p* > 0.05) and 1.06-fold (*p* > 0.05), respectively. However, treatment with an IE_50_ dose of DFB-V significantly reduced the total protein content in both laboratory-reared and wild-caught strains by 2.25-fold (*p* < 0.05) compared to the control. The respective reductions were, however, 2.29-fold and 2.16-fold (*p* < 0.05) when compared to the DFB-treated larvae (Table 2). Note that the wild-caught strains had significantly lower levels of proteins than the laboratory strain, irrespective of the treatment.

**TABLE 2:** Protein content in early fourth instar larvae of *Aedes aegypti* treated with IE_50_ dose of diflubenzuron (DFB) and diflubenzuron + verapamil (DFB-V) (1:10) for 24 h.

Strains of *Ae. aegypti*	Protein content in early fourth instar larvae of *Ae. aegypti* ± SEM (mg/mL)		
	Control	Treatment with DFB at IE_50_ dosage (0.37 µg/L)	Treatment with DFB-V (1:10) at IE_50_ dosage (0.32 µg/L)
Insecticide-susceptible strain (AND-*Ae. aegypti*)	5.265 ± 0.050^a*^	5.361 ± 0.141^a*^ (+1.02)	2.337 ± 0.123^b*^ (−2.25)
Wild-caught strain (GVD*-Ae. aegypti*)	2.162 ± 0.083^a#^	2.294 ± 0.079^a#^ (+1.06)	0.962 ± 0.060^b#^ (−2.25)

IE_50_ refers to the concentration that inhibits 50% of adult emergence. Values with different letters in each row and different symbols in each column are significantly different (*p* < 0.05), one-way ANOVA, followed by Tukey’s all-pairwise multiple comparison tests. Values in brackets refer to fold change from respective control. SEM: standard error of the mean.

### 3.3 GST activity

**FIGURE 3:**
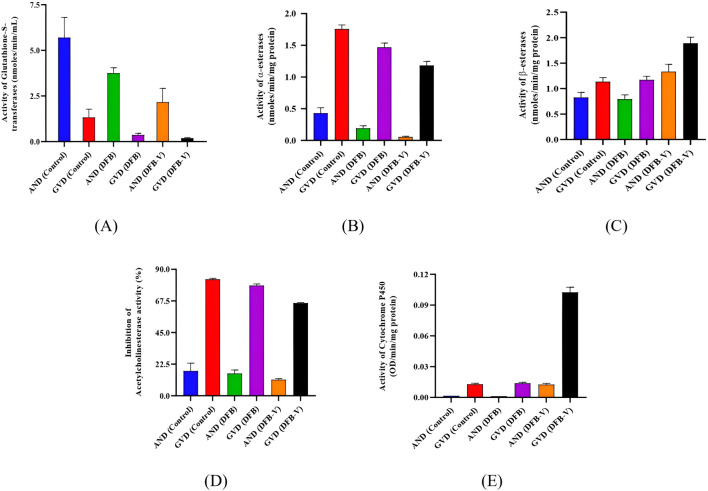
Specific activity of various detoxifying enzymes in the early fourth instars of AND-*Ae. aegypti* and GVD*-Ae. aegypti* strain of *Ae. aegypti* on exposure to IE_50_ of diflubenzuron and diflubenzuron–verapamil (1:10). **(A)** Glutathione S-transferase activity, **(B)** α-esterase activity, **(C)** β-esterase activity, **(D)** percentage acetylcholinesterase inhibition, and **(E)** CYP450 activity. AND = AND-*Ae. aegypti* strain, GVD = GVD-*Ae. aegypti* strain, DFB = Treatment with diflubenzuron, DFB-V = Treatment with diflubenzuron and verapamil (1:10).

The total and specific GST activity was found to be significantly reduced in wild-caught larvae compared to the laboratory-reared larvae, whether untreated or treated (Tables 3–6). Treatment of AND-*Ae. aegypti* larvae with DFB and DFB-V showed significant 1.52-fold and 2.64-fold (*p* < 0.05) decreases in GST activity compared to the control group (Tables 3, 4; Figure 3A), while higher reductions of 3.98-fold and 6.94-fold (*p* < 0.05) were observed in the GVD*-Ae. aegypti* (Tables 5, 6; Figure 3A).

**TABLE 3:** Total activity of detoxifying enzymes in the early fourth instar larvae of the insecticide-susceptible strain of *Aedes aegypti* (AND-*Ae. aegypti*) treated with IE_50_ dosage of diflubenzuron (DFB) and diflubenzuron + verapamil (DFB-V) (1:10) for 24 h.

Treatment	Total activity of detoxifying enzymes				
	Glutathione S-transferase (nmol ± SEM)	α-esterase (mmol ± SEM)	β-esterase (mmol ± SEM)	Acetylcholinesterase (OD ± SEM)	Cytochrome P450 (mmol ± SEM)
Control	0.950 ± 0.189^a^	0.023 ± 0.005^a^	0.044 ± 0.006^a^	0.824 ± 0.056^a^	0.094 ± 0.017^a^
DFB alone	0.625 ± 0.047^b^	0.010 ± 0.002^b^	0.043 ± 0.005^a^	0.841 ± 0.024^a^	0.071 ± 0.005^b^
DFB-V	0.360 ± 0.097^c^	0.001 ± 0.001^c^	0.031 ± 0.002^b^	0.885 ± 0.008^a^	0.323 ± 0.034^c^

Data represent the mean activity in 60 larvae (three replicates of 20 larvae each); SEM: standard error of the mean; IE_50_ refers to the concentration that inhibits 50% of adult emergence. Values with different letters in each column are significantly different (*p* < 0.05), one-way ANOVA, followed by Tukey’s all-pairwise multiple comparison tests.

**TABLE 4:** Specific activity of detoxifying enzymes in the early fourth instar larvae of the insecticide-susceptible strain of *Aedes aegypti* (AND-*Ae. aegypti*) treated with IE_50_ dosage of diflubenzuron (DFB) and diflubenzuron + verapamil (DFB-V) (1:10) for 24 h.

Treatment	Specific activity of detoxifying enzymes				
	Glutathione S-transferase (nmol/min/mL ± SEM)	α-esterase (nmol/min/mg protein ± SEM)	β-esterase (nmol/min/mg protein ± SEM)	Acetylcholinesterase (% inhibition ± SEM)	Cytochrome P450 (OD/min/mg protein ± SEM)
Control	5.703 ± 1.103^a^	0.431 ± 0.088^a^	0.826 ± 0.099^a^	17.629 ± 5.260^a^	0.002 ± 0.000^a^
DFB alone	3.750 ± 0.297^b^	0.200 ± 0.033^b^	0.794 ± 0.083^a^	15.878 ± 2.478^a^	0.001 ± 0.000^b^
DFB-V	2.161 ± 0.754^c^	1.469 ± 0.066^b^	1.334 ± 0.143^b^	11.456 ± 0.769^a^	0.012 ± 0.001^c^

Data represent the mean activity in 60 larvae (three replicates of 20 larvae each); SEM: standard error of the mean; IE_50_ refers to the concentration that inhibits 50% of adult emergence. Values with different letters in each column are significantly different (*p* < 0.05), one-way ANOVA, followed by Tukey’s all-pairwise multiple comparison tests.

**TABLE 5:** Total activity of detoxifying enzymes in the early fourth instar larvae of the wild-caught strain of *Aedes aegypti* (GVD*-Ae. aegypti*) treated with IE_50_ dosage of diflubenzuron (DFB) and diflubenzuron + verapamil (DFB-V) (1:10) for 24 h.

Treatment	Total activity of detoxifying enzymes				
	Glutathione S-transferase (nmol ± SEM)	α-esterase (mmol ± SEM)	β-esterase (mmol ± SEM)	Acetylcholinesterase (OD ± SEM)	Cytochrome P450 (mmol ± SEM)
Control	0.221 ± 0.055^a^	0.038 ± 0.002^a^	0.013 ± 0.002^a^	0.171 ± 0.007^a^	0.311 ± 0.039^a^
DFB alone	0.062 ± 0.014^b^	0.034 ± 0.002^b^	0.040 ± 0.003^a^	0.215 ± 0.011^b^	0.348 ± 0.030^a^
DFB-V	0.032 ± 0.005^c^	0.011 ± 0.001^c^	0.018 ± 0.001^b^	0.341 ± 0.004^c^	1.076 ± 0.07^b^

Data represent mean activity in 60 larvae (three replicates of 20 larvae each); SEM: standard error of the mean; IE_50_ refers to the concentration that inhibits 50% of adult emergence. Values with different letters in each column are significantly different (*p* < 0.05), one-way ANOVA, followed by Tukey’s all-pairwise multiple comparison tests.

**TABLE 6:** Specific activity of detoxifying enzymes in the early fourth instar larvae of the wild-caught strain of *Aedes aegypti* (GVD*-Aedes aegypti*) treated with IE_50_ dosage of diflubenzuron (DFB) and diflubenzuron + verapamil (DFB-V) (1:10) for 24 h.

Treatment	Specific activity of detoxifying enzymes				
	Glutathione S-transferase (nmol/min/mL ± SEM)	α-esterase (nmol/min/mg protein ± SEM)	β-esterase (nmol/min/mg protein ± SEM)	Acetylcholinesterase (% inhibition ± SEM)	Cytochrome P450 (OD/min/mg protein ± SEM)
Control	1.325 ± 0.458^a^	1.758 ± 0.063^a^	1.135 ± 0.081^a^	82.898 ± 0.677^a^	0.013 ± 0.001^a^
DFB alone	0.369 ± 0.087^b^	1.469 ± 0.066^b^	1.171 ± 0.070^a^	78.465 ± 1.113^b^	0.014 ± 0.001^a^
DFB-V	0.191 ± 0.033^c^	1.182 ± 0.066^c^	1.891 ± 0.117^b^	65.951 ± 0.402^c^	0.102 ± 0.005^b^

Data represent mean activity in 60 larvae (three replicates of 20 larvae each); SEM: standard error of the mean; IE_50_ refers to the concentration that inhibits 50% of adult emergence. Values with different letters in each column are significantly different (*p* < 0.05), one-way ANOVA, followed by Tukey’s all-pairwise multiple comparison tests.

### 3.4 α-esterase activity

The laboratory-reared susceptible strain (AND-*Ae. aegypti*) larvae showed a significant (*p* < 0.05) drop of 2.16-fold in α-esterase activity when exposed to diflubenzuron (Tables 3, 4). The activity further reduced to a pronounced 7.70-fold (*p* < 0.05) on treatment with the DFB-V compared to the control group (Figure 3B). On the other hand, the wild-caught strain, which had 4.08-fold higher α-esterase activities than the laboratory strain, showed a comparatively smaller reduction of 1.2-fold (*p* < 0.05) on treatment with DFB alone and a decrease of 1.49-fold (*p* < 0.05) on treatment with DFB-V (Tables 5, 6).

### 3.5 β-esterase activity

Notably, a contrasting augmented effect was observed on the activity of β-esterase in both the tested strains of *Ae. aegypti*. The AND-*Ae. aegypti* larvae showed almost similar activity of 1.04-fold (*p* > 0.05), while an increase of 1.62-fold (*p* < 0.05) in β-esterase activity was found when they were treated with DFB and DFB-V, respectively (Tables 3, 4; Figure 3C). However, the GVD*-Ae. aegypti* strain exhibited almost similar β-esterase activity of 1.03-fold (*p* > 0.05) on DFB treatment but a significant rise of 1.67-fold (*p* < 0.05) on DFB-V treatment. Like α-esterase and β-esterase levels were higher in the wild-caught strain than in the susceptible strain.

### 3.6 Percentage acetylcholinesterase inhibition

The control population of the wild-caught strain of *Ae. aegypti* showed a much higher percentage AChE inhibition than the susceptible strain. The inhibition of AChE activity however, decreased in both strains on larval treatment with DFB and DFB-V (Figure 3D). The AND-*Ae. aegypti* larvae showed a 1.11% and 1.54% (*p* > 0.05) decreased AChE inhibition with DFB and DFB-V treatment (Tables 3, 4). The percentage reductions recorded in GVD*-Ae. aegypti* larvae were 1.06% and 1.26% on treatment with DFB and DFB-V (*p* < 0.05), respectively.

### 3.7 CYP450 monooxygenases

Like esterases, the CYP450 levels were higher in GVD*-Ae. aegypti* larvae than the insecticide-susceptible larvae. The respective treatment of AND-*Ae. aegypti* larvae with DFB and DFB-V registered 1.33-fold reduced but 7.8-fold (*p* < 0.05) enhanced CYP450 monooxygenase activity (Tables 3, 4; Figure 3E). On the other hand, the GVD*-Ae. aegypti* strain exhibited a 1.08-fold (*p* > 0.05) and 8.00-fold (*p* < 0.05) increase in CYP450 activity by respective DFB and DFB-V treatment (Tables 5, 6).

### 3.8 Effect on non-target organisms

The treatment of non-target organisms, *G. affinis, M. thermocyclopoides*, and *P. tetraurelia,* with the respective IE_50_ values of DFB or DFB-V obtained for *Ae. aegypti* early fourth instar did not impart any negative effects on their survival, morphology, and behavior.

## 4 Discussion

The present study was an attempt to examine the possible use of verapamil along with DFB to increase its efficiency against *Ae. aegypti.* The potential of DFB alone as well as in combination with verapamil (1:10) was assessed on the adult emergence and detoxification enzymes of *Ae. aegypti* larvae. The effect was assessed against two strains of dengue vector: a wild-caught GVD*-Ae. aegypti* strain and an insecticide-susceptible AND-*Aedes aegypti* strain maintained in the laboratory to understand their defense system. The compounds were also evaluated on common non-target species.

The larval treatment with DFB significantly inhibited adult emergence in both strains of *Ae. aegypti.* Enhanced inhibition in the adult emergence was obtained with the use of DFB-verapamil (1:10), which indicates the synergistic effect of verapamil on DFB and the plausible use of the mixture to control a mosquito population. The results also showed higher inhibitory effects of DFB and DFB-V against the laboratory-reared susceptible strain compared to the wild-caught strain. This effect may possibly be due to the frequent and indiscriminate use of DFB and other toxicants in the fields, leading to the probable development of some extent of resistance. Furthermore, the higher effects of DFB-V against wild-caught larvae indicated higher synergism and the potential to reduce larval resistance. This could be helpful in managing DFB resistance in the field strains of *Ae. aegypti* and increasing DFB toxicity. The efficacy of DFB against *Ae. aegypti* has been demonstrated by Fansiri et al. (2022), who obtained an IE_50_ value of 2.41 µg/L, which is much higher than that obtained in the present study. Toxicity estimation of DFB against *Anopheles quadrimaculatus* revealed 86.7% larval mortality at 12.5 μg/L (Zhu et al., 2007). The literature, however, reports limited studies that signify the use of verapamil as a synergist of DFB. Porretta et al. (2008) reported efficient synergism of DFB with verapamil against *Ae. caspius*, causing a reduction in the LD_50_ value of diflubenzuron by 16.4-fold. Higher synergism obtained in the study may be ascribed to the variation in species, geographical location, and DFB resistance level of the strain.

The aberrant growth and development caused by exposure to a xenobiotic has been attributed to the altered levels and metabolism of various biochemical constituents present in an organism (Rodríguez-Ortega et al., 2003). The titer of major nutrients, such as proteins, carbohydrates, and fats, essential for an organism’s growth, development, and physiological functions is a good indicator of the metabolic state of organisms (Zhu et al., 2012). The present study estimated the protein levels in the larvae of *Ae. aegypti* treated with DFB/DFB-V to correlate them with the detoxifying enzyme levels.

The results revealed an insignificant increase in total protein in *Ae. aegypti* larvae on treatment with IE_50_ of diflubenzuron, which reduced significantly on DFB-V treatment. This suggests the potential of the verapamil–DFB combination to cause metabolic disruption that likely hindered the larvae’s growth and development. Furthermore, lower protein content in the larvae of the wild-caught strain than the laboratory strain may be due to continual exposure stress of chemicals in the fields. It has been suggested that DFB treatment can cause a decrease in total proteins due to increased biodegradation rates of proteins (Muthusamy et al., 2011), decreased enzyme activity, inhibited DNA synthesis (Hamouda, 2002), or DNA damage that shuts down essential genes responsible for protein production (El-Bermawy and Abulyazid, 1998).

Studies exploring the effects of DFB and DFB-V on the protein reserves of mosquitoes are not available in the literature. However, other IGRs have been tested against different insects, resulting in altered protein levels. Sublethal concentrations of lufenuron have reduced protein levels in *Ae. aegypti* (Panmei et al., 2021), *Pectinophora gossypiella* (Ahmed et al., 2012), and *Glyphodes pyloalis* (Piri Aliabadi et al., 2016). Likewise, inhibition of the protein content was recorded in the hemolymph of the fifth instar nymphs of *Schistocerca gregaria* when treated with three IGRs: pyriproxyfen, tebufenozide, and lufenuron (Ghoneim et al., 2012). In contrast, Linvy et al. (2018) found that application of methoxyfenozide (0.005–1 µg/5 µL acetone) significantly increased the total proteins in the hemolymph of *Spodoptera mauritia* larvae. The contrasting results could be due to variations in the IGR potency, sensitivity of species, immune system, or the treated developmental stage (Ghoneim et al., 2003).

Because all organisms have a defense mechanism to combat external stress by altering the expression of detoxification enzymes, the estimation of the activity of these enzymes becomes crucial to assess the capability of mosquitoes to bear this stress and perform normal physiological functions (Li and Liu, 2007). Hence, DFB- and DFB-verapamil-treated *Ae. aegypti* larvae were evaluated for the detoxification enzymes’ activities. The investigations showed variably increased activities of a few enzymes (β-esterases, CYP450), higher activity of AChE due to decreased inhibition, but a decrease in α-esterase and GST activities in *Ae. aegypti* larvae post-treatment. A higher impact on enzyme activity was observed with DFB-V than DFB alone and on the wild-caught larvae compared to the susceptible strain. It is suggested that higher tolerance to DFB and higher alterations in the enzyme levels of wild-caught strains may be caused by the changes in the activity of detoxifying enzymes already induced by the stress caused by other chemicals applied in the fields.

The present investigations showed the probable involvement of β-esterases, AChE, and CYP450 in detoxifying DFB. These observations are aligned with the reports of Anwar and Abd El-Mageed (2005), who found that diflubenzuron increased β-esterase activity in cotton leafworms, *Spodoptera littoralis,* while decreasing α-esterase activity. In contrast, Hamdy and Azab (2002) found that chlorfluazuron and hexaflumuron increased α-esterase enzyme activity in *S. littoralis* while decreasing β-esterase enzyme levels. However, increased levels of both non-specific esterases have been reported in *Ae. aegypti* after lufenuron treatment (Panmei et al., 2021) and in *Spodoptera litura* after methoxyfenozide treatment (Wang et al., 2009). Suppressed esterase levels have been observed in *S. litura* on exposure to the sublethal doses of lufenuron, tebufenozide, and flufenoxuron (Bakr et al., 2013; Ismail, 2020).

The significant decrease in the GST activity in both the AND-*Ae. aegypti* and GVD*-Ae. aegypti* strains on treatment with diflubenzuron and its synergized form indicates its non-involvement in detoxification of DFB. These results are in accordance with the studies performed in *S. littoralis* larvae exposed to lufenuron and chlorfluazuron (Abou-Taleb et al., 2015). They reported 38.6% and 45.6% suppressed GST activity in the larvae on respective treatment with 0.28 ppm and 0.62 ppm lufenuron. On the other hand, Panmei et al. (2021) showed a noticeable rise in GST activity in *Ae. aegypti* treated with lufenuron, indicating its possible role in lufenuron detoxification.

Unlike GST activity, 24 h of treatment with diflubenzuron increased CYP450 activity in the *Ae. aegypti* larvae of both the investigated strains, suggesting an effective and instant activation of the detoxification mechanism. In addition, decreased percentage inhibition of AChE in the treated larvae with respect to the control indicates the probable role of AChE in DFB detoxification. Similar observations have been recorded by Panmei et al. (2021) on lufenuron treatment of *Ae. aegypti* larvae, revealing a decreased percentage AChE inhibition and a rise in CYP450 levels. A similar rise in monooxygenases has been shown in *Lucilia cuprina,* the blowfly, on DFB exposure (Kotze et al., 1997). In contrast, lufenuron treatment (at LC_25_ level) increased the percentage AChE inhibition in *S. littoralis* larvae (Ismail, 2020). It is apparent that the lufenuron toxicity in *Ae. aegypti* larvae plausibly blocked the action potential in the neurons, leading to AChE inhibition.

The present results have shown that DFB alone or in combination with verapamil at IE_50_ values did not affect the survival of non-target organisms, *G. affinis, M. thermocyclopoides*, and *P. tetraurelia*. Numerous reports have suggested the safe use of DFB in the environment being non-toxic to non-target organisms, though a few studies have reported their toxic effects on fishes and invertebrates during acute and chronic exposures (Farlow et al., 1978; Abe et al., 2019; Moe et al., 2019). The impact assessment of DFB against aquatic insects—*Corixa punctata* and *Notonecta glauca—*and crustaceans—*Anisops sardea, Plea minutissima*, and *Daphnia magna*—revealed significant toxic effects on *C. punctata* and medium toxic effects on the rest (Seeradj et al., 2022). The toxic effects of DFB, however, depend upon the exposure duration, dosage used, and sensitivity of organisms.

The present study demonstrated the efficacy of DFB against laboratory and field strains of *Ae. aegypti* at very low dosages (0.37 µg/L; 0.63 µg/L), which were further reduced by the use of verapamil (0.32 µg/L; 0.46 µg/L). These doses are significantly low compared to the dosages recommended by WHO (0.25 mg/L) in potable water, which thus signifies DFB’s safe use in the environment.

## 5 Conclusion

The present study showed the effective use of the diflubenzuron–verapamil combination against *Ae. aegypti* larvae, which caused higher adult emergence inhibition than diflubenzuron alone. The higher effects of the DFB-V combination obtained against the wild strain indicate its efficient use for mosquito management. In addition, the differential activities of detoxifying enzymes in DFB- and DFB-V-treated *Ae. aegypti* larvae and the higher impacts obtained with DFB-V and on the wild-caught larvae propose the plausible use of verapamil along with diflubenzuron for imparting higher efficacy. Moreover, the non-toxicity of DFB and DFB-V against non-target organisms indicates their safe use in the environment.

## Data Availability

The original contributions presented in the study are included in the article/supplementary material; further inquiries can be directed to the corresponding author.

## References

[B1] Abe F. R. Machado A. A. Coleone A. C. da Cruze C. Machado-Neto J. G. (2019). Toxicity of diflubenzuron and temephos on freshwater fishes: ecotoxicological assays with *Oreochromis niloticus* and *Hyphessobrycon eques* . Water Air Soil Pollut. 230, 77. 10.1007/s11270-019-4128-7

[B2] Abou-Taleb H. K. Zahran H. M. Gad A. A. (2015). Biochemical and physiological effects of lufenuron and chlorfluazuron on *Spodoptera littoralis* (Boisd.) (Lepidoptera: Noctuidae). J. Entomol. 12 (2), 77–86. 10.3923/je.2015.77.86

[B3] Ahmed A. F. Moustafa H. Z. kandi M. (2012). Toxicological and biochemical studies of lufenuron, chlorfluazuron and chromafenozide against *Pectinophora gossypiella* (Saunders). Acad. J. Biol. Sci. C Physiol. Mol. Biol. 4 (1), 37–47. 10.21608/eajbsf.2012.17281

[B4] Anwar E. M. Abd El-mageed A. E. M. (2005). Toxicity impacts of certain insect growth regulators on some biochemical activities of the cotton leafworm. J. Agric. Res. 83 (3), 915–935. 10.17221/3/2F2011-PPS

[B5] Bakr R. F. Abd Elaziz M. F. El-Barky N. M. Awad M. H. El-Halim A. Hisham M. E. (2013). The activity of some detoxification enzymes in *Spodoptera littoralis* (Boisd.) larvae (Lepidoptera–Noctuidae) treated with two different insect growth regulators. Acad. J. Biol. Sci. C. Physiol. Mol. Biol. 5 (2), 19–27. 10.21608/EAJBSC.2013.16092

[B6] Belinato T. A. Valle D. (2015). The impact of selection with diflubenzuron, a chitin synthesis inhibitor, on the fitness of two Brazilian *Aedes aegypti* field populations. PloS One 10 (6), e0130719. 10.1371/journal.pone.0130719 26107715 PMC4481264

[B7] Bharati M. Saha D. (2018). Multiple insecticide resistance mechanisms in primary dengue vector, *Aedes aegypti* (Linn.) from dengue endemic districts of sub-Himalayan West Bengal, India. PloS One 13 (9), e0203207. 10.1371/journal.pone.0203207 30199543 PMC6130861

[B8] Borase H. P. Patil C. D. Salunkhe R. B. Narkhede C. P. Salunke B. K. Patil S. V. (2013). Photosynthesized silver nanoparticle: a potent mosquito biolarvicidal agent. J. Nanomed. Biotherapeutic Discov. 3, 1–7. 10.4172/2155-983X.1000111

[B9] Bradford M. M. (1976). A rapid and sensitive method for the quantitation of microgram quantities of protein utilizing the principle of protein-dye binding. Anal. Biochem. 72 (1-2), 248–254. 10.1006/abio.1976.9999 942051

[B10] Brogden W. G. Barber A. M. (1987). Microplate assay of acetylcholinesterase inhibition kinetics in single mosquito homogenates. Pestic. Biochem. Physiol. 29 (3), 252–259. 10.1016/0048-3575(87)90155-6

[B11] Brogden W. G. Barber A. M. (1990). Microplate assay of glutathione-S-transferase activity for resistance detection in single mosquito triturates. Comp. Biochem. Physiol. B 96 (2), 339–342. 10.1016/0305-0491(90)90385-7 2361364

[B12] Brogden W. G. Dickinson C. M. (1983). A microassay system for measuring esterase activity and protein concentration in small samples and in high-pressure liquid chromatography eluate fractions. Anal. Biochem. 131 (2), 499–503. 10.1016/0003-2697(83)90204-x 6614483

[B13] Chen C. D. Seleena B. Chiang Y. F. Lee H. L. (2008). Field evaluation of the bioefficacy of diflubenzuron (Dimilin) against container-breeding *Aedes* sp. mosquitoes. Trop. Biomed. 25 (1), 80–86. 18600208

[B14] Doucet D. Retnakaran A. (2012). Insect chitin: metabolism, genomics and pest management. Adv. Insect Physiol. 43, 437–511. 10.1016/B978-0-12-391500-9.00006-1

[B15] El-Bermawy S. M. Abulyazid I. (1998). Genomic DNA polymorphism and biochemical assay in the pupal stage of Med-fly *Ceratitis capitata* (Wied). Proc. Intern. Conf. Mol. Gent. 1, 165–180.

[B16] Eltahir W. A. Elamin M. O. Mahgoub I. Faraj E. A. (2018). Efficacy of temephos and diflubenzuron use in malaria vector larvae control. World J. Adv. hlthcare. Res. 2 (5), 36–39. 10.1234/OJSDJ.V1I1.6

[B17] Enayati A. A. Ranson H. Hemingway J. (2005). Insect glutathione transferases and insecticide resistance. Insect Mol. Biol. 14 (1), 3–8. 10.1111/j.1365-2583.2004.00529.x 15663770

[B18] Epis S. Porretta D. Mastrantonio V. Comandatore F. Sassera D. Rossi P. (2014). ABC transporters are involved in defense against permethrin insecticide in the malaria vector *Anopheles stephensi* . Parasit. Vectors 7, 349–357. 10.1186/1756-3305-7-349 25073980 PMC4124152

[B19] Fansiri T. Pongsiri A. Khongtak P. Nitatsukprasert C. Chittham W. Jaichapor B. (2022). The impact of insect growth regulators on adult emergence inhibition and the fitness of *Aedes aegypti* field populations in Thailand. Acta Trop. 236, 106695. 10.1016/j.actatropica.2022.106695 36122761

[B21] Farlow J. E. Breaud T. P. Steelman C. D. Schilling P. E. (1978). Effects of the insect growth regulator difluhenzuron on non-target aquatic populations in a Louisiana intermediate marsh. Environ. Entomol. 7 (2), 199–204. 10.1093/ee/7.2.199

[B22] Ghoneim K. S. Al-Dali A. G. Abdel-Ghaffar A. A. (2003). Effectiveness of lufenuron (CGA-184699) and diofenolan (CGA-59205) on the general body metabolism of the red palm weevil, *Rhynchophorus ferrugineus* (Curculionidae: Coleoptera). Pak. J. Biol. Sci. 6 (13), 1125–1129. 10.3923/pjbs.2003.1125.1129

[B23] Ghoneim K. S. Hamadah K. S. Tanani M. A. (2012). Protein disturbance in the haemolymph and fat body of the desert locust *Schistocerca gregaria* as a response to certain insect growth regulators. Bull. Environ. Pharmacol. Life Sci. 1 (7), 73–83.

[B24] Grigoraki L. Puggioli A. Mavridis K. Douris V. Montanari M. Bellini R. (2017). Striking diflubenzuron resistance in *Culex pipiens*, the prime vector of West Nile Virus. Sci. Rep. 7 (1), 11699. 10.1038/s41598-017-12103-1 28916816 PMC5601912

[B25] Guz N. Cagatay N. S. Fotakis E. A. Durmusoglu E. Vontas J. (2020). Detection of diflubenzuron and pyrethroid resistance mutations in *Culex pipiens* from Muğla, Turkey. Acta Trop. 203, 105294. 10.1016/j.actatropica.2019.105294 31836282

[B26] Hamdy A. M. Azab A. M. (2002). “Effect of insect growth regulators and binary mixtures on enzymes activity of Egyptian cotton leafworm, *Spodoptera littoralis*, (Boisd.) larvae,” in 2nd International conference of Plant Protection, Research Institute, Cairo, Egypt, 617–623.

[B27] Hamouda L. S. (2002). Toxicological and biochemical studies on the effect of admiral (IGR) and nuclear polyhedrosis virus (SNPV) on *Spodoptera littoralis* (Boisd.) larvae. J. Egypt. Acad. Soc. Environ. Dev. 2 (1), 15–29. 10.21608/eajbsa.2013.13361

[B28] Ismail S. M. (2020). Effect of sublethal doses of some insecticides and their role on detoxification enzymes and protein-content of *Spodoptera littoralis* (Boisd.) (Lepidoptera: Noctuidae). Bull. Natl. Res. Cent. 44 (1), 1–6. 10.1186/s42269-020-00294-z

[B29] Kang X. L. Zhang M. Wang K. Qiao X. F. Chen M. H. (2016). Molecular cloning, expression pattern of multidrug resistance associated protein 1 (MRP1, ABCC1) gene, and the synergistic effects of verapamil on toxicity of two insecticides in the bird cherry‐oat aphid. Arch. Insect Biochem. Physiol. 92 (1), 65–84. 10.1002/arch.21334 27110952

[B30] Karunaratne S. H. P. P. De Silva W. A. P. P. Weeraratne T. C. Surendran S. N. (2018). Insecticide resistance in mosquitoes: development, mechanisms and monitoring. Ceylon J. Sci. 47 (4), 299–309. 10.4038/cjs.v47i4.7547

[B31] Kona M. P. Kamaraju R. Donnelly M. J. Bhatt R. M. Nanda N. Chourasia M. K. (2018). Characterization and monitoring of deltamethrin-resistance in *Anopheles culicifacies* in the presence of a long-lasting insecticide-treated net intervention. Malar. J. 17 (1), 414. 10.1186/s12936-018-2557-1 30409140 PMC6225645

[B32] Kotze A. C. Sales N. Barchia I. M. (1997). Diflubenzuron tolerance associated with monooxygenase activity in field strain larvae of the Australian sheep blowfly (Diptera: Calliphoridae). J. Econ. Entomol. 90 (1), 15–20. 10.1093/jee/90.1.15 9071887

[B33] Kumar S. Thomas A. Sahgal A. Verma A. Samuel T. Pillai M. K. K. (2002). Effect of synergist, Piperonyl Butoxide, on the development of deltamethrin resistance in yellow fever mosquito, *Aedes aegypti* L. (Diptera: Culicidae). Arch. Insect Biochem. Physiol. 50, 1–8. 10.1002/arch.10021 11948970

[B34] Kumar S. Thomas A. Sahgal A. Verma A. Samuel T. Pillai M. K. K. (2004). Variations in the insecticides resistance spectrum of *Anopheles stephensi* Liston on selections with deltamethrin and deltamethrin/PBO combination. Ann. Trop. Med. Parasitol. 98, 861–871. 10.1179/000349804X3180 15667718

[B35] Li X. Z. Liu Y. H. (2007). Diet influences the detoxification enzyme activity of *Bactrocera tau* (Walker) (Diptera: Tephritidae). Acta Entomol. Sin. 50 (10), 989–995. 10.16380/J.KCXB.2007.10.009

[B36] Linvy V. Sridhu P. Reshma R. M. Resmitha C. Kannan V. M. (2018). Storage protein in the hemolymph of 6th instar larvae of *Spodoptera mauritia* Boisd. (Lepidoptera: Noctuidae) is increased by the ecdysone mimic, methoxyfenozide. Int. J. Entomol. Res. 3 (2), 1–4.

[B37] Lucchesi V. Grimaldi L. Mastrantonio V. Porretta D. Di Bella L. Ruspandini T. (2022). Cuticle modifications and over-expression of the chitin-synthase gene in diflubenzuron-resistant phenotype. Insects 13 (12), 1109. 10.3390/insects13121109 36555019 PMC9782986

[B38] Moe S. J. Hjermann D. Ravagnan E. Bechmann R. K. (2019). Effects of an aquaculture pesticide (diflubenzuron) on non-target shrimp populations: extrapolation from laboratory experiments to the risk of population decline. Ecol. Model. 413, 108833. 10.1016/j.ecolmodel.2019.108833

[B39] Muthusamy M. Shivakumar S. Karthi K. Ramkumar R. (2011). Pesticide detoxifying mechanism in field population of *Spodoptera litura* (Lepidoptera: Noctuidae) from South India. *Egypt* . Acad. J. Biol. Sci. C Physiol. Mol. Biol. 3 (1), 51–57. 10.21608/eajbsf.2011.17437

[B40] Panmei K. Samal R. R. Lanbiliu P. Kumar S. (2021). Influence of lufenuron on the nutrient content and detoxification enzyme expression in *Aedes aegypti* L. (Diptera: Culicidae). Int. J. Trop. Insect Sci. 41 (4), 2965–2973. 10.1007/s42690-021-00481-z

[B41] Pešić B. Kulišić Z. Teodorović R. Trailović S. M. Djokić V. Djordjevic M. (2022). Comparison of mosquito larvicidal formulations of diflubenzuron on *Culex pipiens* mosquitoes in belgrade, Serbia. Acta Vet. 72 (1), 87–99. 10.2478/acve-2022-0007

[B42] Piri Aliabadi F. Sahragard A. Ghadamyari M. (2016). Lethal and sublethal effects of a chitin synthesis inhibitor, lufenuron, against *Glyphodes pyloalis* Walker (Lepidoptera: Pyralidae). J. Crop Prot. 5 (2), 203–214. 10.18869/MODARES.JCP.5.2.203

[B43] Porretta D. Fotakis E. A. Mastrantonio V. Chaskopoulou A. Michaelakis A. Kioulos I. (2019). Focal distribution of diflubenzuron resistance mutations in *Culex pipiens* mosquitoes from Northern Italy. Acta Trop. 193, 106–112. 10.1016/j.actatropica.2019.02.024 30825446

[B44] Porretta D. Gargani M. Bellini R. Medici A. Punelli F. Urbanelli S. (2008). Defence mechanisms against insecticides temephos and diflubenzuron in the mosquito *Aedes caspius*: the P‐glycoprotein efflux pumps. Med. Vet. Entomol. 22 (1), 48–54. 10.1111/j.1365-2915.2008.00712.x 18380653

[B45] Rodríguez-Ortega M. J. Grøvisk B. E. Rodriguez-Ariza A. Goksøyr A. López-Barea J. (2003). Changes in protein expression benefits in bivalve molluscs (*Chamaelea gallina*) exposed to four model environmental pollutants. Proteomics 3 (8), 1535–1543. 10.1002/pmic.200300491 12923779

[B46] Sankar M. Kumar S. (2023). A systematic review on the eco-safe management of mosquitoes with diflubenzuron: an effective growth regulatory agent. Acta Ecol. Sin. 43, 11–19. 10.1016/j.chnaes.2021.09.019

[B47] Seeradj N. Bendali-Saoudi F. Soltani N. (2022). The effect of diflubenzuron (Dimilin® 25 WP) on some non-target aquatic insect and crustacean species. Pol. J. Entomol. 91 (4), 174–183. 10.5604/01.3001.0016.1930

[B48] Wang J. Tian D. Zhuang J. (2009). Selection and risk assessment of *Spodoptera litura* (Fabricius) resistance to methoxyfenozide. Jiang. J. Agric. Sci. 25 (1), 79–83.

[B49] Warikoo R. Kumar S. (2013). Impact of *Argemone mexicana* extracts on the cidal, morphological, and behavioural response of dengue vector, *Aedes aegypti* L. (Diptera: Culicidae). Parasitol. Res. 112 (10), 3477–3484. 10.1007/s00436-013-3528-7 23835923

[B51] World Health Organization (1998). Techniques to detect insecticide resistance mechanisms (field and laboratory manual). Geneva: WHO. Available at: https://apps.who.int/iris/handle/10665/83780.

[B56] World Health Organization (2005). Guidelines for laboratory and field testing of mosquito larvicides. Geneva: WHO. Available at: https://apps.who.int/iris/handle/10665/69101.

[B50] World Health Organisation (2024). Fact sheets. Dengue and severe dengue. Geneva: WHO. Available at: https://www.who.int/news-room/fact-sheets/detail/dengue-and-severe-dengue (Accessed January, 2024).

[B52] Yao R. Zhao D.-D. Zhang S. Zhou L.-Q. Wang X. Gao C.-F. (2017). Monitoring and mechanisms of insecticide resistance in *Chilo suppressalis* (Lepidoptera: crambidae), with special reference to diamides. Pest Manag. Sci. 73 (6), 1169–1178. 10.1002/ps.4439 27624654

[B53] Zhu K. Y. Heise S. Zhang J. Anderson T. D. Starkey S. R. (2007). Comparative studies on effects of three chitin synthesis inhibitors on common malaria mosquito (Diptera: Culicidae). J. Med. Entomol. 44 (6), 1047–1053. 10.1603/0022-2585(2007)44[1047:csoeot]2.0.co;2 18047205

[B54] Zhu Q. He Y. Yao J. Liu Y. Tao L. Huang Q. (2012). Effects of sublethal concentrations of the chitin synthesis inhibitor, hexaflumuron, on the development and hemolymph physiology of the cutworm, *Spodoptera litura* . J. Insect Sci. 12 (1), 27. 10.1673/031.012.2701 22958164 PMC3472920

[B55] Zibadee A. Zibaee I. Sendi J. J. (2011). A juvenile hormone analog, pyriproxyfen, affects some biochemical components in the hemolymph and fat bodies of *Eurygaster integriceps* Puton (Hemipter: scutelleridae). Pestic. Biochem. Physiol. 100, 289–298. 10.1016/j.pestbp.2011.05.002

